# Forest and steppe communities of Kazakh Uplands: vegetation plot dataset

**DOI:** 10.3897/BDJ.14.e189416

**Published:** 2026-08-17

**Authors:** Natalya Ivanova, Margarita Ishmuratova, Sayagul Tyrzhanova

**Affiliations:** 1 Institute of Mathematical Problems of Biology RAS – the Branch of Keldysh Institute of Applied Mathematics of Russian Academy of Sciences, Pushchino, Russia Institute of Mathematical Problems of Biology RAS – the Branch of Keldysh Institute of Applied Mathematics of Russian Academy of Sciences Pushchino Russia; 2 Karaganda National Research University named after аcademician Ye.A. Buketov, Karaganda, Kazakhstan Karaganda National Research University named after аcademician Ye.A. Buketov Karaganda Kazakhstan

**Keywords:** *Acer negundo*, Bektauata Mountains, Buiratau State National Natural Park, Karkaraly State National Natural Park, Ortau Mountains, Saryarka, Ulytau State National Natural Park, vegetation relevés

## Abstract

**Background:**

Kazakhstan is usually associated with steppes; only 5% of the territory is covered by forests. The forest areas are small and located in the Altai and Trans-Ili Alatau mountains, as well as the northern border of the country. Forest "oases" are also present in Central Kazakhstan. These are unique and almost unexplored relict forest sites, historically linked to the boreal forests of Altai and Siberia. These forests are relatively small and placed in isolated areas of the Kazakh Upland mountain ranges, surrounded by vast steppe landscapes. Until now, vegetation data from Central Kazakhstan (especially forest habitats) are still limited in digital repositories. Therefore, the aim of this work was to summarise, standardise and publish the vegetation relevés collected in Central Kazakhstan.

**New information:**

The presented dataset is the first open-access relevé collection that contributes to the knowledge of the current vegetation of the Kazakh Uplands, Central Kazakhstan. It provides 117 vegetation plots (3473 associated occurrences) sampled in 2014 and 2022–2025. The data cover two IUCN nature-protected areas (Karkaraly and Buiratau State National Natural Parks), Ulytau State National Natural Park and the Ortau and Bektauata Mountains, as well as ecologically valuable nature areas without protected status. Data on invasive vascular plant species occurrences are provided for the first time.

## Introduction

Kazakhstan, the world’s largest landlocked country, spans central Eurasia. It is bordered by Russia, China and the Central Asian countries, exhibiting a pronounced geographical gradient in its topography. This diversified terrain system fosters rich flora and fauna as well as unique types of natural communities (Convention on Biological Diversity 2025). In recent years, increasingly more data on the plant biodiversity of Kazakhstan have become available through the Global Biodiversity Information Facility (GBIF). The digitisation of regional herbarium collections has begun and ~ 30,000 specimens are already available through the GBIF (Imanbayeva et al. 2023; Issakova and Lagus 2023; Ishmuratova et al. 2023; Romanchuk 2024; Sultangazina and Baitleva 2025). The contribution of citizen scientists to digital biodiversity data is growing. In May 2026, the number of iNaturalist observations in Kazakhstan exceeded 300,000, of which more than 172,000 were of vascular plants. Despite this, the available relevé data on Kazakhstan are still limited. The GrassPlot repository (Biurrun et al. 2019) includes 200 vegetation surveys of steppe communities. A total of 151 relevés of abandoned croplands and steppes is deposited in the Global Index of Vegetation-Plot Databases (Mathar et al. 2015). The forests of Kazakhstan have remained outside the scope of plant scientists until now. At the same time, these local forest communities are of great scientific interest. The Zenodo dataset presented in this article (Ivanova et al. 2026) fills the gap in the data on the vegetation of the relict boreal forests of Central Kazakhstan and also contains some data on the steppe communities of this region.

## Sampling methods

### Study extent

**Figure 1.**
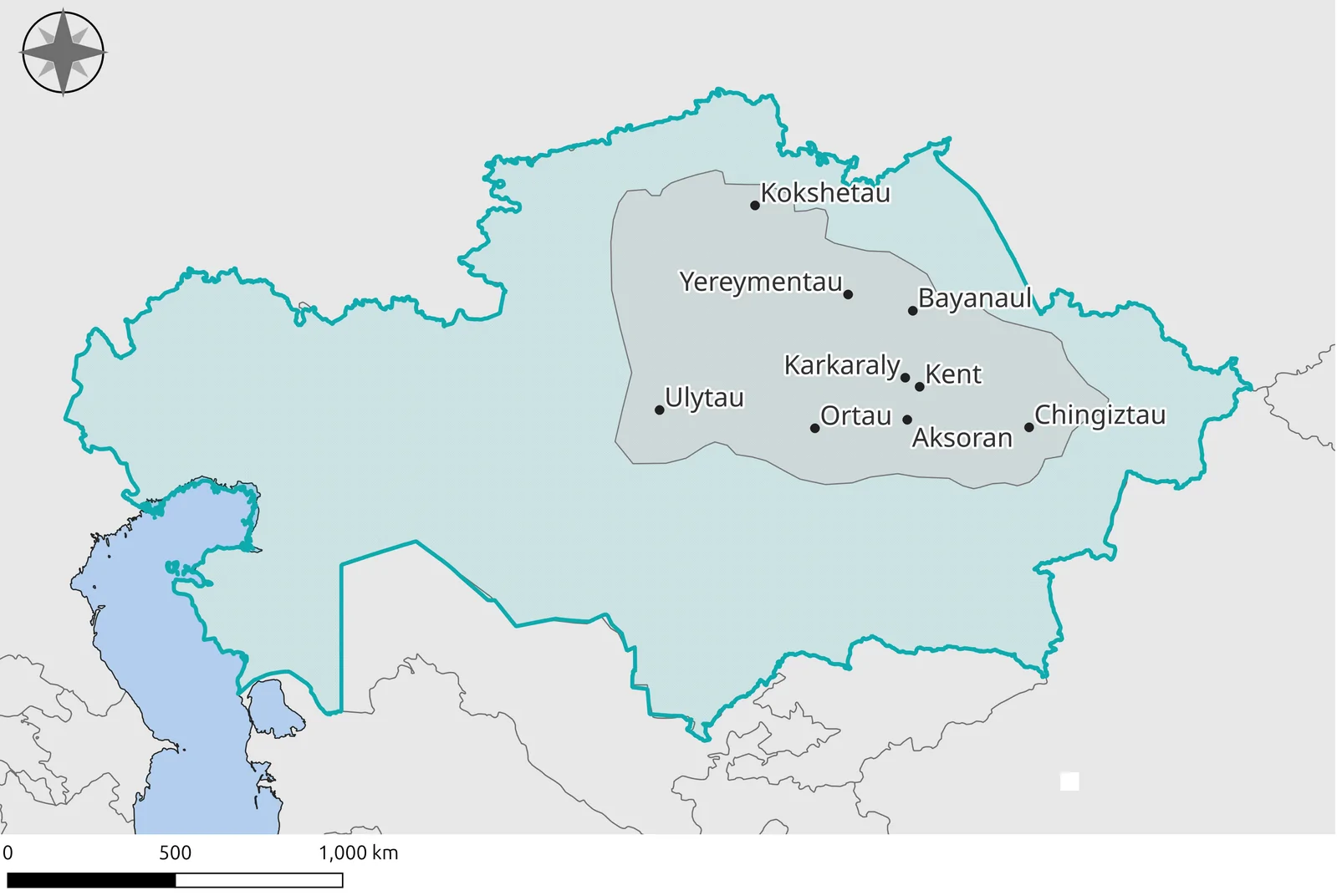
Main mountain ranges of the Kazakh Uplands (grey background area).

The Kazakh Uplands (Saryarqa) are located east of the Turgay Plateau and represent an elevated, undulating plain with separate low mountain ranges (Fig. 1). The western part extends 950 km in a meridional direction, has average absolute heights of 300 to 500 m and is characterised by a very flat relief. The Ulytau (1133 m) and Kokshetau (947 m) mountain ranges are located here. The eastern part runs north to south for 350–400 km and is characterised by high absolute elevations and a more rugged terrain. In this part of the Uplands are the Karkaraly Mountains (1403 m), Aksoran (1565 m) and Chingiztau (1305 m) (Gvozdecky and Mikhailov 1987). The climate of this area is sharply continental, with hot, temperate summers and cold, snowy winters. The average temperature in winter is about -13°C, in summer about +24°C and annual precipitation is 180–250 mm (Maksutova et al. 2005). Steppe and semi-desert vegetation prevails (Rachkovskaya 2016). Forests cover less than 2% of the area (Dancheva and Zalesov 2024) and are represented by relatively small "steppe kolki" (small forested areas in the steppe, usually dominated by birch trees) and larger mountain-forest areas, which are relicts of a formerly continuous forest territory connected with the forests of Western Siberia and Altai during the cold and wet Pleistocene epochs. Due to this, they have preserved a complex of boreal (and other forest) plant species considerably distant from the main range (Gorchakovskii 1987).

### Sampling description

The dataset provides 117 relevés collected by our team in 2014 and 2022–2025 during different research projects. Each project is assigned a code (see dwc: datasetName in the dataset); the summary of each project is presented in Table 1. The PermanentPlots project summarises vegetation surveys on permanent monitoring plots in natural sites of the Karkaraly State National Natural Park. Relevés of communities with high grazing pressure (Bisons project) were collected in the recreational part of the Park in the animal pen. The Scabiosa project was aimed at studying *Scabiosa ochroleuca* L. and *Lomelosia isetensis* (L.) Soják populations (Tyrzhanova and Silanteva 2021). CentralKZ relevés were sampled in forest communities of Central Kazakhstan during the study of wild fruit plants (Ivanova and Shashkov 2025). Data for the Kendara project were collected in strongly protected forests of the Karkaraly State National Natural Park to study invasions of alien plant species and earthworms (Lumbricidae).

Vegetation surveys were carried out according to the relevé method (Mueller-Dombois and Ellenberg 1974, Chytrý and Otýpková 2003) using the Braun-Blanquet or Drude cover-abundance scale. The total cover and individual cover by species for all vascular plants were estimated for the following vegetation layers (if present): the tree canopy layer (A); the understorey layer, including tree undergrowth and tall shrubs in forests or shrubs in steppes (B); and the herb layer (C) (Serebryakov 1962). Sub-layers A1 and B1 were indicated to layers A and B, as necessary. All sampling plots were georeferenced in the WGS84 datum. Locations of relevés are shown in the Geographic coverage section.

**Figure 2.**
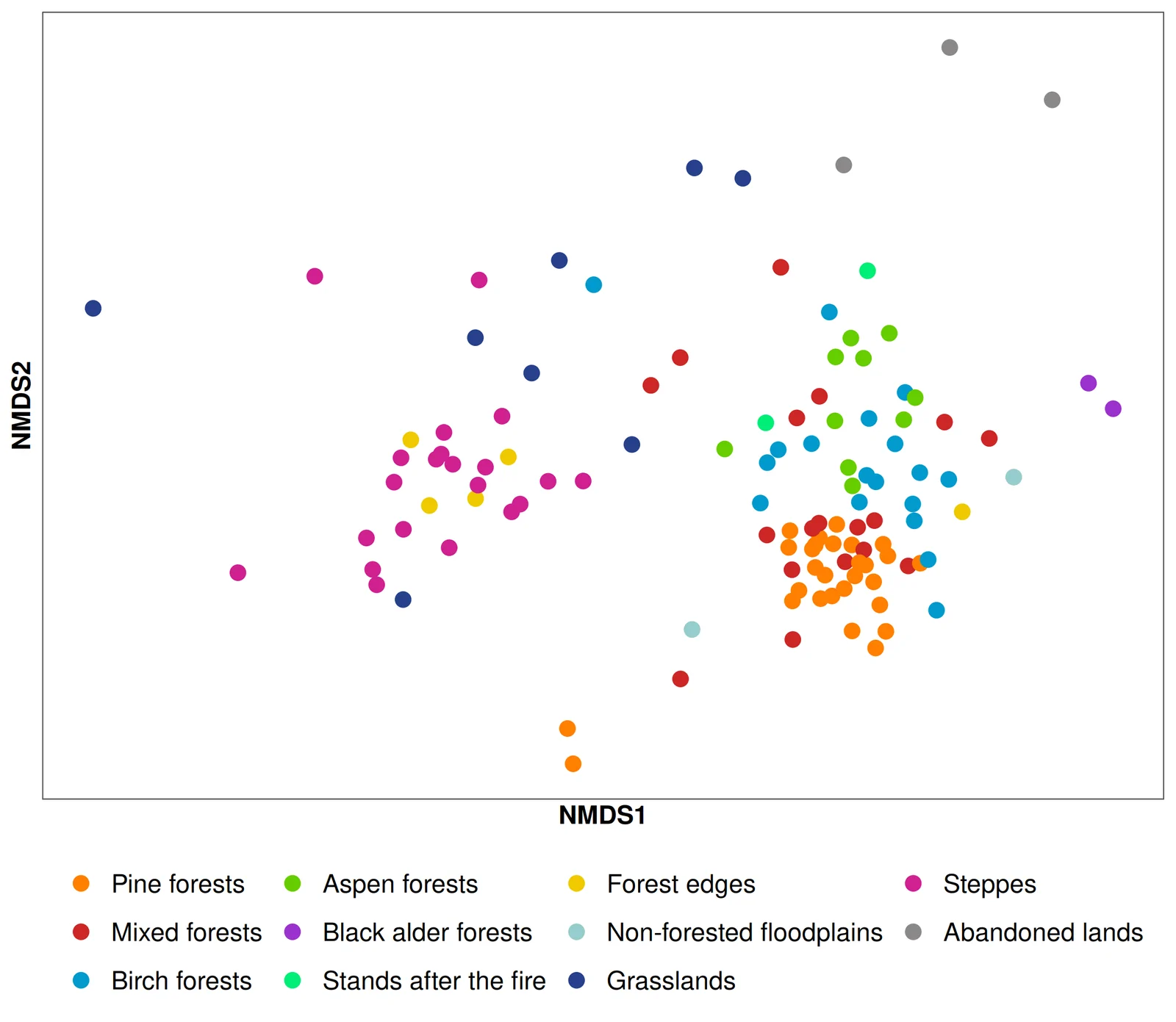
NMDS ordination of vegetation relevés of Central Kazakhstan.

Our data cover different habitat types (Fig. 2). Most of the relevés (76 plots) were collected in forest stands, two plots in forest sites after fires and five in forest edges; 21 surveys were made in steppe (dry xerophytic forb, forb–feather grass steppes and forb-grass meadow steppes types according to Rachkovskaya and Bragina (2012)) and eight in grassland (grass-forb type according to Rachkovskaya and Bragina (2012)) habitats, two plots in floodplains and three on abandoned agricultural lands. Pine-dominated tree stands (*Pinus sylvestris* L.) were more present in forest relevés (28 plots); the other relevés were made in birch (*Betula pendula* Roth), aspen (*Populus tremula* L.) and mixed stands. Only two vegetation surveys were carried out in black alder stands; *Alnus glutinosa* (L.) Gaertn. is a protected species in Kazakhstan (Baytulin 2014) and communities with black alder are rare, probably due to the economic use of this tree.

### Quality control

To control the quality of the data, we followed basic principles (Chapman 2017). Species were identified by Prof. Margarita Ishmuratova. She is a specialist in botany and has wide experience in floristic studies of this region (Ishmuratova et al. 2016, Ishmuratova et al. 2024). Species identification is carried out using "Flora of the USSR" (Komarov 1964), "Flora of Kazakhstan" (Pavlov 1966), "Keys of Vascular Plants of Karkaraly National Park" (Kuprijanov et al. 2008), "Flora of Siberia" (Krasnoborov 2003), "Keys of Plants of Kemerovo Region" (Krasnoborov 2001) and "Keys of Plants of Central Asia" (Institute of Botany 1993). Herbarium specimens were deposited in the QAR collection. Scientific names were checked using the GBIF species matching tool.

### Step description

Field data were digitised and standardised according to the Darwin Core (Wieczorek et al. 2012). At the first step, relevés were published through GBIF as sampling event datasets (Ishmuratova and Ivanova 2024, Ishmuratova and Ivanova 2025, Ishmuratova et al. 2025, Tyrzhanova et al. 2025, Ivanova et al. 2026). The GBIF Relevé Darwin Core Extension was used for providing data about the cover of vegetation layers. Currently, information from this extension is not indexed by GBIF. Therefore, in the second step, all relevés were merged and published through Zenodo (Ivanova et al. 2026) to provide full access to collected data.

## Geographic coverage

### Description

**Figure 3.**
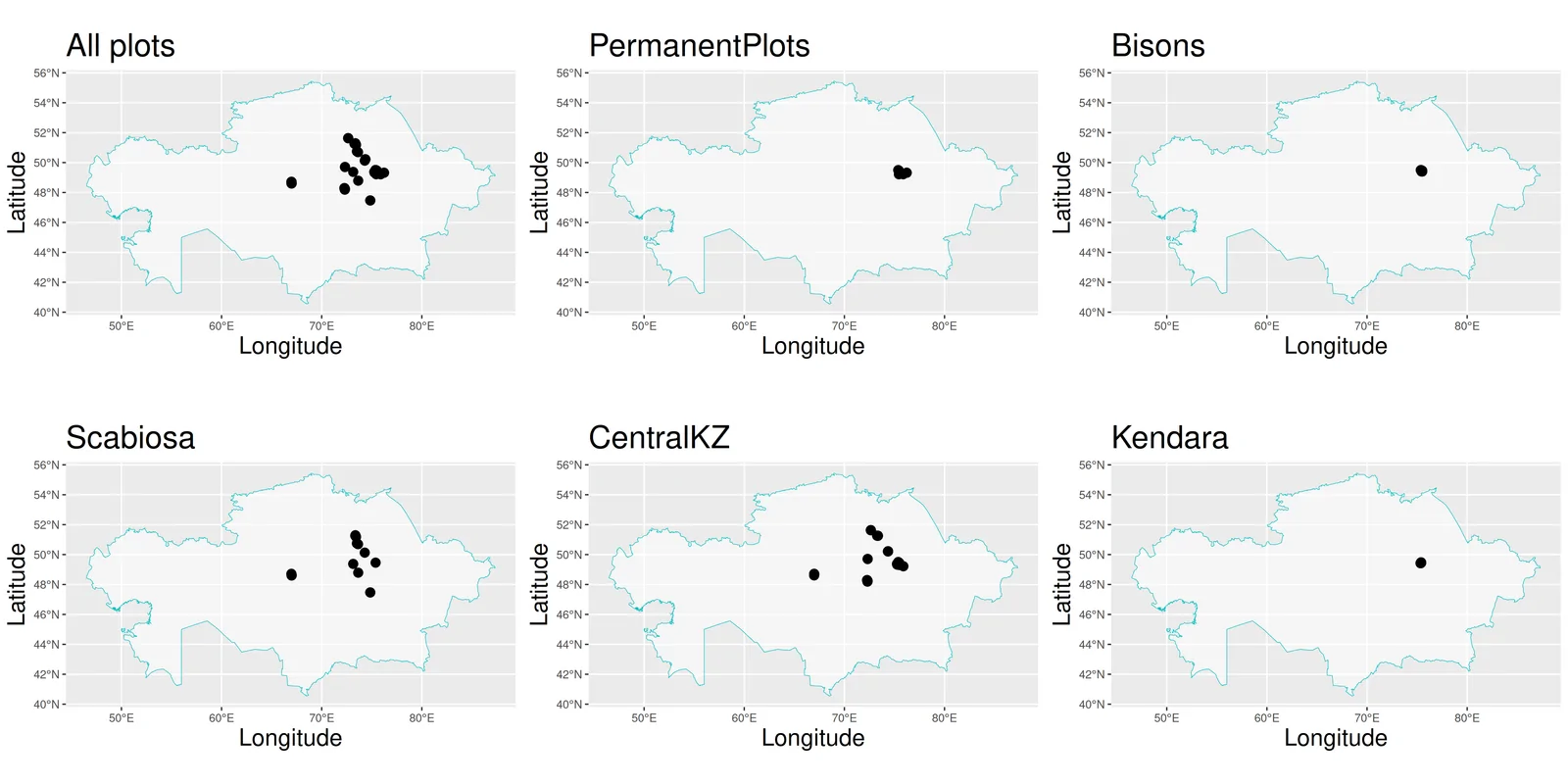
The spatial scope of projects contributing to the dataset.

Our data have been collected from the territory of Central Kazakhstan (Qaraghandy and Ulytau Regions) and partly from the southern part of Akmola Region (Northern Kazakhstan) (Fig. 3). The presented data differ in geographic coverage. The most wide-ranging coverage is provided by the Scabiosa and CentralKZ projects, which include relevés from Karkaraly State National Natural Park, Ortau Mountain forests, Buiratau State National Natural Park, Ulytau State National Natural Park, Bektauata Mountain and steppe kolki from different areas of the Qaraghandy Region. Data from PermanentPlots, Bisons and Kendara projects were collected in the Karkaraly State National Natural Park.

### Coordinates

47.46957 and 51.63357 Latitude; 66.963623 and 76.22422 Longitude.

## Taxonomic coverage

### Description

**Figure 4.**
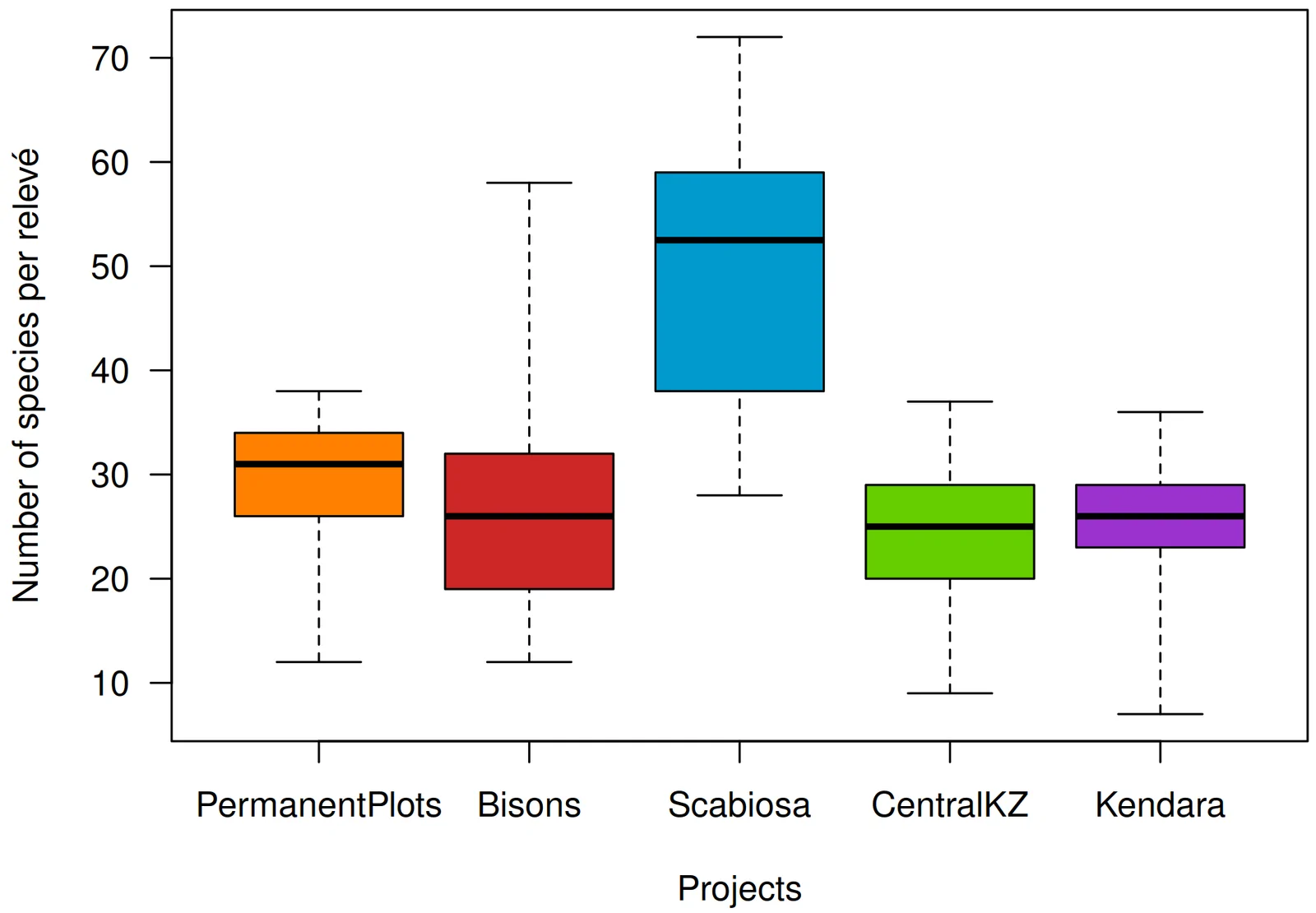
Species diversity in projects contributing to the dataset.

The dataset includes 481 unique scientific names (dwc: scientificName); 60 families and 238 genera were recorded. Amongst them, 420 taxa were identified to the species level, 12 to subspecies and three to varieties. The largest number of species per relevé was recorded in the Scabiosa project, which is explained by the large size of the sample plots (Fig. 4). Amongst the 100 m^2^ plots, more species were recorded in the PermanentPlots project because it included not only forest, but also forest edge habitats, where species diversity is usually rich.

Presented relevés expand available data about the distribution of alien plant species. Today, Kazakhstan (like other countries) faces significant issues with alien species (Kovshar 2020, Sitpaeva et al. 2023). However, in Central Asian countries, alien plants until recently were considered part of floristic accounts and checklists and, therefore, have not been the subject of a separate study at the national scale. To this time, only Kyrgyzstan has a national checklist of alien plant species (Sennikov and Lazkov 2021). In Kazakhstan, an inventory of invasive species was carried out in the Almaty, Jetisu and Ulytau Regions (Khusainova et al. 2023; Yeszhanova et al. 2025). Therefore, our data contribute to the development of a national list of invasive species.

The presented vegetation relevés provide the occurrences of eight alien species: *Agropyron cristatum* (L.) Gaertn., *Echinops sphaerocephalus* L., *Echium vulgare* L., *Cota tinctoria* (L.) J.Gay, *Malus baccata* (L.) Borkh., *Caragana arborescens* Lam., *Stellaria media* (L.) Vill. and *Acer negundo* L. The characteristics of these species are based on the results of previous studies of the flora of Central Kazakhstan (Ishmuratova et al. 2024) and are presented in a dataset in accordance with the study (Groom et al. 2019) (see dwc: establishmentMeans, dwc: degreeOfEstablishment and dwc: pathway fields). *Agropyron cristatum* was introduced in the Qaraghandy Region at the end of the 18^th^ and 19^th^ centuries as a forage culture. At first, *Agropyron cristatum* was grown on a large scale for hay production, but later the plant became widespread in the steppe zone of Central and Northern Kazakhstan. *Echinops sphaerocephalus* was introduced in the Qaraghandy Region as a decorative culture from Eastern Kazakhstan, now partially grown in natural populations. *Echium vulgare*, previously not registered in natural flora, possibly was introduced along with the other cultural species. Currently, this species is spreading through areas distributed by human activity or carried by the wind. *Cota tinctoria* and *Malus baccata* were introduced in Central Kazakhstan as decorative and fruit-berry plants. *Caragana arborescens* was introduced in this region and is used for landscaping, creating green hedges, afforestation and soil stabilisation. It can reproduce to a limited extent in natural communities. *Stellaria media* was introduced via soil and potted plants with a closed root system. In Central Kazakhstan, it is a common weed found in fields and vegetable gardens. In natural habitats, this species is recorded in moist sites.

In our opinion, *Acer negundo* (Boxelder maple) is the most dangerous amongst recorded invasive plants. In Russia, *Acer negundo* is listed amongst the 100 most dangerous invasive species (Dgebuadze et al. 2018). It is widely distributed across the country, from the Kaliningrad Oblast to the Primorsky Krai. This species colonises railroad embankments, river floodplains, city parks and natural forests, forming monospecific tree stands and suppressing native plant species (Vinogradova et al. 2022). In Central Asian countries, *Acer negundo* has been widely used in urban landscaping since the 1870s (Vinogradova 2006). Recent studies (Yeszhanova et al. 2025) and our unpublished field data show the beginning of colonisation in natural communities in Central Kazakhstan. In our vegetation relevés, *Acer negundo* was recorded twice. The first occurrence was located in the Ortau Mountain forests, where it was found in the aspen tree stand. The total cover of the B layer was 25%, dominated by *Acer negundo*, *Rosa majalis* Herrm., *Salix rosmarinifolia* L. and *Lonicera tatarica* L. The herb layer was dominated by *Calamagrostis epigejos* (L.) Roth, *Poa nemoralis* L. and *Geum urbanum* L. The second occurrence was located in the Karkaraly State National Natural Park. One young individual of *Acer negundo* was found in the mixed forest stand (B layer). The herb layer in this site was dominated by *Melica nutans* L., *Galium boreale* L. and *Brachypodium pinnatum* (L.) P. Beauv.

## Temporal coverage

**Formation period:** 2014, 2022-2025.

### Notes

Bisons relevés were collected in 2014, PermanentPlots, and Scabiosa data were sampled in 2022; CentralKZ relevés were collected in 2023 and 2024; and Kendara data were sampled in 2025.

## Usage licence

### Usage licence

Other

### IP rights notes

CC-BY

## Data resources

### Data package title

Vegetation relevés Kazakh Uplands

### Resource link

https://doi.org/10.5281/zenodo.20523471

### Alternative identifiers

https://zenodo.org/records/20523471

### Number of data sets

1

### Data set 1.

#### Data set name

Vegetation relevés Kazakh Uplands

#### Data format

Darwin Core

#### Character set

UTF-8

#### Description

The dataset consists of two tables. The event table describes sampling plot characteristics (location, date of the surveys, methods, cover of vegetation layers etc.). The occurrence table contains species abundance data.

**Data set 1.** 

Column label	Column description
eventID	An identifier for the relevé.
decimalLatitude	The geographic latitude in decimal degrees.
decimalLongitude	The geographic longitude in decimal degrees.
geodeticDatum	The ellipsoid upon which the geographic coordinates given in dwc:decimalLatitude and dwc:decimalLongitude are based ('WGS84' in this dataset).
coordinateUncertaintyInMeters	The horizontal distance (in metres) from the given dwc:decimalLatitude and dwc:decimalLongitude describing the smallest circle containing the whole of the location.
coordinatePrecision	A decimal representation of the precision of the coordinates given in the dwc:decimalLatitude and dwc:decimalLongitude.
eventDate	Date of the vegetation survey in YYYY-MM-DD format.
year	Year of the vegetation survey.
month	Month the vegetation survey.
day	Day the vegetation survey.
country	The name of the country ('Kazakhstan' in this dataset).
countryCode	The standard code for the country according to ISO 3166-1-alpha-2 ('KZ' in this dataset).
stateProvince	The name of the next smaller administrative region than country ('Qaraghandy Region', 'Ulytau Rgion' or 'Akmola Region' in this dataset).
county	The full, unabbreviated name of the next smaller administrative region than stateProvince.
locality	The specific description of the place where the relevé was made.
verbatimLocality	The original textual description of the place where the relevé was made.
habitat	Original description of the habitat.
samplingProtocol	The name method used during a dwc:Event ('vegetation relevé' in this dataset).
sampleSizeValue	A numeric value of the vegetation sampling plot (100 or 1600 in this dataset).
sampleSizeUnit	The unit of measurement of the size of the vegetation sampling plot ('m^2^' in this dataset).
language	A language of the record.
coverTreesInPercentage	The cover (%) of trees (A layer).
coverShrubsInPercentage	The cover (%) of undergrowth and shrubs (B layer).
coverHerbsInPercentage	The cover (%) of herbs (C layer).
datasetID	GBIF identifier of the corresponding dataset.
datasetName	Code of the project ('PermanentPlots', 'Bisons', 'Scabiosa', 'CentralKZ' or 'Kendara' in this dataset).
rightsHolder	The organisation owning and managing rights over the resource ('Karaganda National Research University named after аcademician Ye.A. Buketov' in this dataset).
occurrenceID	An identifier for the dwc:Occurrence.
eventID	An identifier for the relevé.
occurrenceRemarks	Vegetation layer.
scientificName	The full scientific name according to the GBIF Backbone.
verbatimScientificName	The full scientific name according to field data.
organismQuantity	The abundance of the species.
organismQuantityType	The name of abundance scale ('Drude Scale' or 'Braun-Blanquet Scale' in this dataset).
basisOfRecord	The specific nature of the data record ('HumanObservation' in this dataset).
occurrenceStatus	A statement about the presence or absence of the species ('present' in this dataset).
taxonRank	The taxonomic rank of the most specific name in the dwc:scientificName.
kingdom	The full scientific name of the kingdom ('Plantae' in this dataset).
family	The full scientific name of the family.
taxonID	An identifier for the Taxon mentioned in the dwc: scientificName according to the GBIF Backbone.
recordedBy	A list of people contributed to this record.
recordedByID	A list of ORCIDs for people mentioned in the dwc: recordedBy.
establishmentMeans	Statement about whether the species has been introduced to a given place and time through the direct or indirect activity of modern humans.
degreeOfEstablishment	The degree to which this species survives, reproduces and expands its range at the given place and time.
pathway	The process by which the species came to be in a given place at a given time.

## Figures and Tables

**Table 1.** The scope of projects contributed to the dataset

Project code	Number of vegetation plots	Number of species occurrences	Abundance scale	Sampling plot size, m^2^
PermanentPlots	9	264	Drude scale	100
Bisons	16	429	Drude scale	100
Scabiosa	14	687	Braun-Blanquet Scale	1600
CentralKZ	41	1028	Braun-Blanquet Scale	100
Kendara	37	1065	Braun-Blanquet Scale	100
In total	117	3473		
